# The Efficacy of Er:YAG Laser in the Extraction of Impacted Third Molars: A Randomized Clinical Trial

**DOI:** 10.3390/dj12120388

**Published:** 2024-11-27

**Authors:** Diana Sologova, Ekaterina Diachkova, Susanna Sologova, Elena Smolyarchuk, Arus Margaryan, Ekaterina Grigorevskikh, Pavel Petruk, Elizaveta Tumanova, Oxana Svitich, Svetlana Tarasenko

**Affiliations:** 1 Department of Oral Surgery of the Institute of Dentistry, I.M. Sechenov First Moscow State Medical University (Sechenov University), 119048 Moscow, Russia; dyachkova_e_yu_1@staff.sechenov.ru (E.D.); mistelizaveta@yandex.ru (E.T.); tarasenko_s_v@staff.sechenov.ru (S.T.); 2 Department of Pharmacology, Nelyubin Institute of Pharmacy, I.M. Sechenov First Moscow State Medical University (Sechenov University), 119991 Moscow, Russia; sologova_s_s@staff.sechenov.ru (S.S.); smolyarchuk_e_a@staff.sechenov.ru (E.S.); margaryan_a_g@staff.sechenov.ru (A.M.); grigorevskikh_e_m@staff.sechenov.ru (E.G.); 3 Maxillofacial Surgery Department, I.M. Sechenov First Moscow State Medical University (Sechenov University), 119991 Moscow, Russia; petruk_p_s@staff.sechenov.ru; 4 Department of Microbiology, Virology, and Immunology, I.M. Sechenov First Moscow State Medical University (Sechenov University), 125009 Moscow, Russia; svitich_o_a@staff.sechenov.ru; 5 I.I. Mechnikov Research Institute of Vaccines and Sera, 105064 Moscow, Russia

**Keywords:** Er:YAG laser, impacted third molar, extraction, laser bone cutting

## Abstract

**(1) Background**: Impacted third molar extraction with a scalpel and rotary instruments is one of the most traumatic surgeries in dentistry. Therefore, it is necessary to discover less traumatic methods and instruments to reduce the risk of postoperative complications. **(2) Methods**: This study is reported in accordance with the CONSORT guidelines. The study aim is to assess the effectiveness of an Er:YAG laser with a wavelength of 2.94 μm, cutting and rotating instruments in the extraction of lower third molars in comparison with the traditional instruments using clinical and radiology parameters. In the control group, the impacted third molars were extracted with the traditional instruments, like scalpel and rotary instruments; in the test group, the impacted third molars were extracted with an Er:YAG laser. As per the inclusion and exclusion criteria, we enrolled 60 patients who were randomly assigned into two groups (Er:YAG laser group and control group). The efficacy of the Er:YAG laser was assessed by postoperative pain, collateral swelling, mouth opening, and radiology parameters such as radiographic infrabony defects and radiographic bone height after tooth extraction. **(3) Results**: The results showed that the clinical postoperative parameters like pain, collateral swelling, and mouth opening were less pronounced in the Er:YAG laser group than those in the control group (*p* < 0.001). According to the data of the radiology parameters (RBH and RID), the regeneration of the socket after extraction was better in the laser group than in the control group (*p* < 0.001). **(4) Conclusions**: Based on the obtained results of clinical and radiology parameters assessment, it was shown that third molar extraction using an Er:YAG laser is a less traumatic method than extraction using a scalpel and rotary instruments.

## 1. Introduction

Impacted lower third molars are the most common pathology among dento-mandibular anomalies that result in the malposition of the third molars [1]. The impaction of the third molars in the oral cavity may lead to complications such as caries and the development of periodontal disease [2]. The extraction of third molars accounts for about 90% of all elective surgical procedures, and it is one of the most common surgeries performed by oral and maxillofacial surgeons [3,4].

Third molar extraction is accompanied by a number of postoperative complications due to the highly traumatic nature of the operation. According to the literature, the range of complication rates after the extraction of impacted third molars can vary from 5% to 31% [5]. Complications after the extraction of lower third molars alongside antibiotic therapy have different frequencies of occurrence. In 3.4% of cases, there was a local inflammatory reaction in the area of the extracted tooth; in 3.8%, there was a “dry hole”; in 1.9%, the patients had a fever; and in 15%, they had trismus [6,7].

In order to systematize the variants of third molar positioning, several classifications of third molar placement have been developed [8,9]. In the modern scientific literature and in clinical practice, the following classifications are most often used: Winter, Tetsch and Wagner, Pell and Gregory, and Asanami and Kasazaki [10,11,12,13,14,15]. Asanami and Kasazaki in 1993 created a classification that takes into account both the angulation and the spatial position of the molar in relation to the occlusal plane and the anterior margin of the mandibular branch. This classification describes the degree of retention of the lower third molar vertically and horizontally, the inclination of the third molar relative to the axis of the second molar, and the location of the retained tooth relative to the anterior margin of the mandibular branch [10]. The classifications of impacted lower third molars make it possible to determine the degree of retention of the tooth, which makes it possible to determine the degree of difficulty of the procedure preoperatively and to plan a surgical intervention [16,17].

Currently, there is a need to search for minimally invasive methods of tissue alteration for more comfort during the postoperative period. Nowadays, many techniques and drug administration options exist in modern medicine. Some of them feature a preoperative single dose of prednisone in third molar surgery, using the piezosurgery technique and dexamethasone injection, and therapeutic elastic bandage application [18,19,20]. Laser technologies are one of the methods for atraumatic surgical intervention. The main properties of laser technologies include that they are less traumatic, minimally invasive, painless, and result in a favorable postoperative period [21,22,23,24,25].

Laser radiation has a number of both surgical and therapeutic effects; that is why it has been widely applied in periodontology, implantology, maxillofacial surgery, the treatment of oral mucosa diseases and inflammatory diseases, endodontics, caries treatment, and teeth whitening [23,26,27]. High-intensity erbium-based laser technologies (Er:YAG and Er, Cr:YSGG) have become widespread in dental surgery due to their ability to work on both soft and hard tissues, such as enamel, tooth dentin, and bone tissue [22,28,29,30,31,32].

Our study aim is to carry out a comparative analysis of the effectiveness of using an Er:YAG laser with a wavelength of 2.94 μm, cutting and rotating instruments during the extraction of lower third molars according to the data of clinical and radiology parameters. The primary endpoint of our study is the measurement of radiographic bone height (RBH) after 6 months after extraction.

The null hypothesis of our study is there are no differences between the postoperative clinical and radiological parameters in the test and control groups.

## 2. Materials and Methods

### 2.1. Study Design

Between June 2023 and June 2024, 60 subjects were enrolled in a single-blind, randomized, controlled clinical trial with a parallel design of two independent groups with a 1:1 allocation ratio; these were outpatients at the Clinic of Oral Surgery of the Faculty of Dental Medicine I.M. Sechenov First Moscow State Medical University (Sechenov University), Moscow, Russia. This study was registered in the Registry of Clinical Trials and followed the guidelines described in the CONSORT 2010 (Consolidated Standards of Reporting Trials) statement on clinical trials. Registration number: NCT05540015. Blinding was achieved with blinded radiologists.

After applying the sample size formula, we determined 52 patients [33]. We added 10% of dropout and obtained 60 patients.

**Figure 1:**
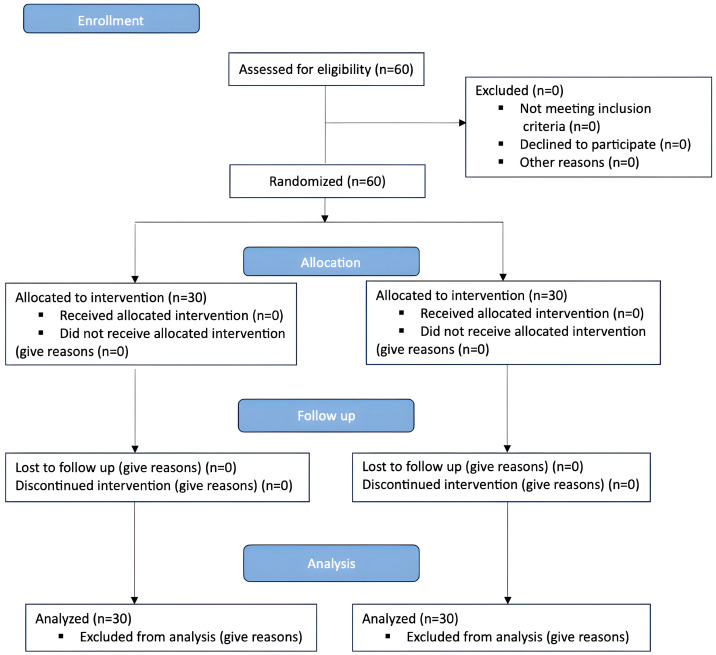
CONSORT flow chart.

All examined patients were divided into 2 groups (Figure 1).

Group 1 included 30 patients who underwent surgery to extract impacted mandibular third molars using the traditional method with cutting and rotary instruments.

Group 2 included 30 patients who underwent surgery to extract impacted mandibular third molars using an Er:YAG laser with a wavelength of 2940 nm.

Clinical and radiology (X-ray) parameters were assessed before and after the extraction of the impacted third molars.

### 2.2. Patient Treatment Characteristics

Treatment of patients was performed at the Department of Surgical Dentistry of the Clinical Centre of the Borovsky Institute of Dentistry. E.V. Borovsky I.M. Sechenov First Moscow State Medical University (Sechenov University) of the Ministry of Health of the Russian Federation. A total of 60 patients with diagnosis of impacted teeth (K01.0) and (K01.1), who were indicated for the extraction of impacted lower third molars, were examined.

According to the inclusion and non-inclusion criteria, 60 patients aged from 21 to 70 years were included in the study.

The inclusion criteria are outlined below:

(1) Age of patients from 21 to 70 years;
(2) Indications for extraction of impacted lower third molars;
(3) Presence of the mandibular second molar in the dental arch;
(4) Absence of concomitant pathologies or concomitant pathologies in the stage of compensation;
(5) Therapeutic and surgical sanitation of the oral cavity;
(6) Satisfactory oral hygiene;

The exclusion criteria are outlined below:

(1) Non-compliance by age group;
(2) Presence of concomitant pathologies in the stage of exacerbation and decompensation;
(3) Pregnancy, lactation period;
(4) Mental illnesses;
(5) Malignant neoplasms;
(6) Alcohol and drug abuse in the anamnesis;
(7) Poor oral hygiene;
(8) Absence of the second molars in the dental arch;
(9) Inflammatory periodontal diseases;

The withdrawal criteria are outlined below:

(1) Exacerbation of concomitant pathology during the study;
(2) Violation of doctor’s recommendations, deviation from the study protocol;
(3) Patient’s unwillingness to continue participation in the study.

#### 2.2.1. Method of Third Molar Extraction Using Erbium Laser with Wavelength 2940 nm

**Figure 2:**
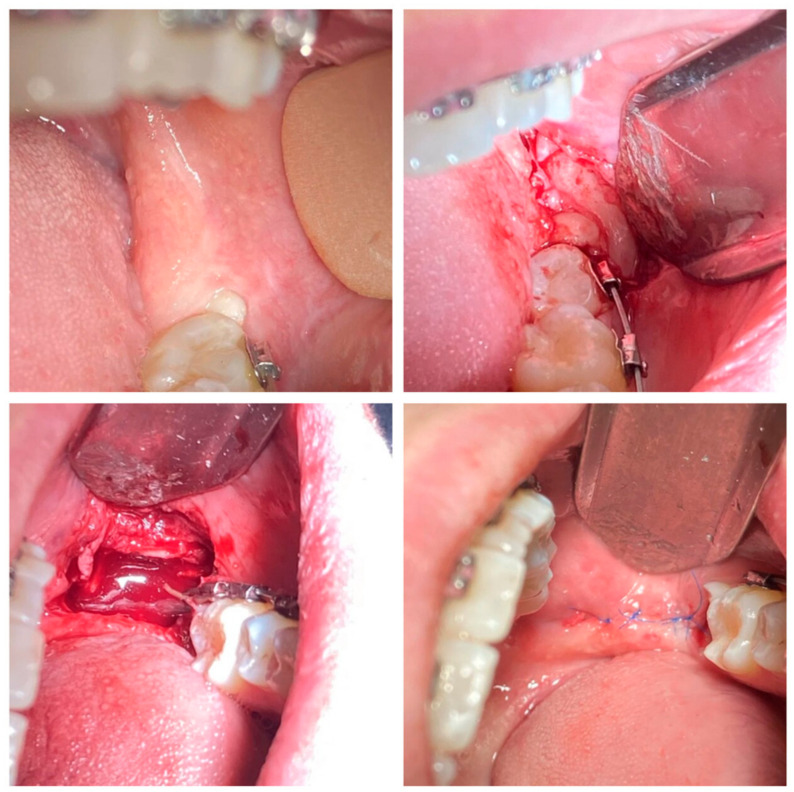
Method of third molar extraction using erbium laser with wavelength 2940 nm.

Patients from the study group 2 with the following diagnoses: retained teeth (K01.0), impacted teeth (K01.1), chronic apical periodontitis (K04.5), and cementum caries (K02.2)—had their lower third molars extracted using the 2940 nm wavelength erbium laser (Figure 2). Under local anesthesia with Sol. Ultracaini DS Forte 1:100,000, Sanofi, Berlin, Germany, an atraumatic incision was made with an erbium laser (DEKA, Smart 2940D plus, Er:YAG laser system, Firenze, Italy) beam in the ablation mode: at an energy of 100 mJ, pulse frequency of 10 Hz, with light contact of the fibers with the mucosal surface, without pressure, at an angle of 45. The diameter of the laser beam is 1 mm (at handpieces output); water and air spray were used.

The L-shaped flap in the area of the impacted third molar was peeled off using a rasp. A laser beam in a very short mode (short pulses—230 µs) with the energy of 150 mJ and pulse frequency of 10 Hz was used to perform ostectomy of the overhanging edge of the bone tissue above the third molar; if necessary, the tooth was separated into parts, increasing the energy up to 200 mJ and pulse frequency up to 20 Hz. Using the elevators and luxators, the tooth was extracted from the cavity. Afterwards, we performed revision of the extracted tooth well, control of the integrity of the well walls, and hemostasis. The flap was placed in the initial position, and the wound was sutured with simple knotted sutures using a non-resorbable monofilament suture material, polypropylene 5/0.

#### 2.2.2. Traditional Method of Third Molar Extraction

**Figure 3:**
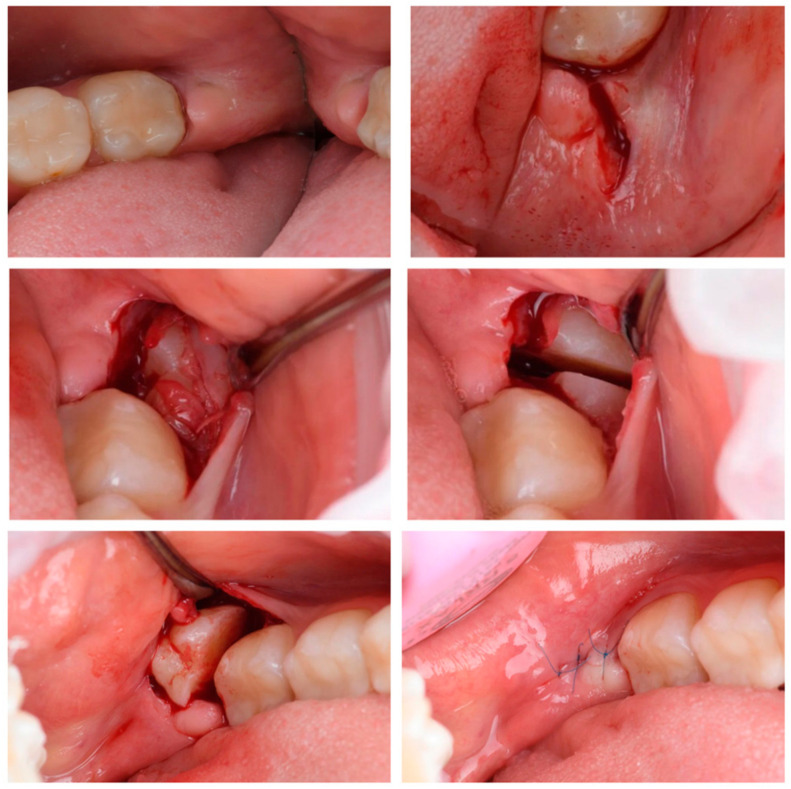
Traditional method of third molar extraction.

Patients in study group 1 with diagnoses of retained teeth (K01.0), impacted teeth (K01.1), chronic apical periodontitis (K04.5), and cementum caries (K02.2) had their lower third molars extracted using cutting instruments and a rotary instrument group (Figure 3). Under local anesthesia with Sol. Ultracaini DS Forte 1:100,000, a 15C scalpel was used to make an incision, and a mucosal–adcostal L-shaped flap was peeled off in the area of the retained mandibular third molar. Using a turbine handpiece, a Lindemann surgical bur, elevators and luxators, the third molar was extracted. Afterwards, we performed a revision of the extracted tooth, control of the integrity of the cavity walls, and hemostasis. The flap was mobilized, placed in the initial position, and the wound was sutured with simple knotted sutures using non-resorbable monofilament suture material: polypropylene 5/0.

#### 2.2.3. Clinical Parameters of the Postoperative Period After Extraction of Third Molars

Postoperative clinical examination of patients was on days 1, 3, 5, 7 and 10 after surgery. The patient’s postoperative pain, collateral swelling and mouth opening were assessed.

##### Postoperative Pain

Postoperative pain was assessed on days 1, 3, 5, 7 and 10 using the 11-point visual analogue scale (VAS) [34]. This is a subjective method of pain assessment, which is a 10-point scale: 0 points—no pain, 1 to 3 points—mild pain, 4 to 6 points—medium pain, 7 to 9 points—severe pain, 10 points—very severe pain. When entering data into the table, the number of painkillers taken was taken into account.

##### Collateral Swelling

**Figure 4:**
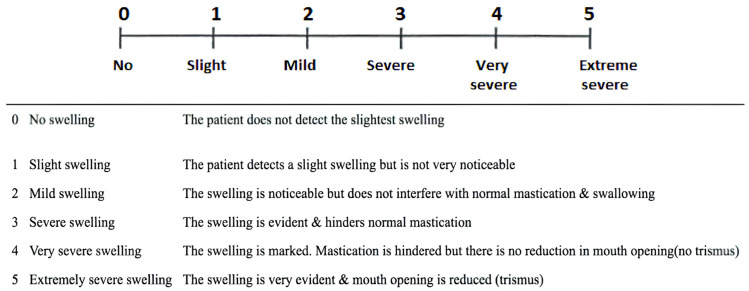
Collateral swelling assessment tool.

Assessment of collateral swelling of soft tissues was performed on days 1, 3, 5, 7 and 10 after surgery on a 5-point scale, where 0 points—no soft tissue swelling, 1 point—patient feels weak insignificant swelling of tissues, 2 points—noticeable small swelling that does not complicate chewing and swallowing movements, 3 points—noticeable soft tissue swelling that affects chewing and swallowing, 4 points—strong soft tissue swelling that does not cause muscle contracture, 5 points—very strong soft tissue swelling that causes muscle contracture (Figure 4). At each postoperative examination, the data on collateral edema in the form of scores were entered into the table.

##### Mouth Opening

Mouth opening was assessed before surgery on days 1, 3, 5, 7 and 10 of the postoperative period. Measurements were made with a caliper with the mouth as open as possible in millimeters (mm). The distance was measured with a caliper from the cutting edge of the maxillary central incisor to the cutting edge of the mandibular central incisor. Measurement data were entered into the table in millimeters (mm).

##### Hyperesthesia

Hyperesthesia of the distal root of the second molar was assessed after the healing of soft tissues in the extraction area. The presence or absence of hyperesthesia was assessed with binary criterion (1/0).

#### 2.2.4. Radiology Parameters

**Figure 5:**
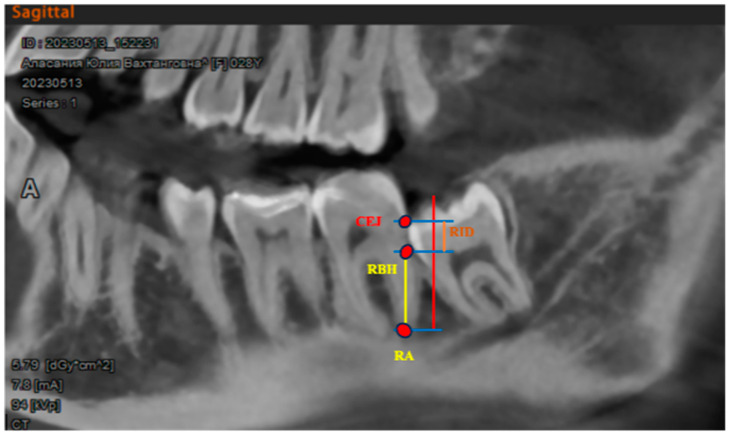
Radiology parameters: radiographic infrabony defect (RID); radiographic bone height (RBH); root apex (RA); cementoenamel junction (CEJ).

Radiology parameters such as “radiographic infrabony defect” (RID) and “radiographic bone height” (RBH) were analyzed on CVCT [33] (Figure 5). Parameters were examined immediately after the extraction at 12 weeks and 24 weeks to assess the bone regeneration process.

The radiographic bone height (RBH) was determined as the distance between the uppermost point, where the M2 distal root and the mesial wall of extraction socket intersected, and the root apex (RA). The radiographic infrabony defect (RID) was determined as the distance from the RBH to the cementoenamel junction to evaluate bone regeneration within the socket.

### 2.3. Ethical Considerations

All patients signed an informed consent form for surgical intervention before surgical treatment and were informed in detail about the timing and stages of dental treatment. Ethical Committee approval was obtained for all studies (extract from the minutes of the Ethical Committee meeting № 20-21 dated 18 November 2021).

### 2.4. Statistics

All obtained research data were summarized in statistical tables, which was followed by calculations, computations and statistic evaluation. The jamovi project (2024) (Version 2.5) Sydney, Australia was used for statistic evaluation.

The primary endpoint was the radiographic bone height (RBH) at 6 months after extraction. The secondary endpoints included radiographic infrabony defect (RID), clinical parameters such as postoperative pain, collateral swelling and mouth opening.

The randomization process was conducted by the envelope method.

The postoperative pain, collateral swelling and mouth opening were assessed by the Mann–Whitney statistical method (intergroup comparisons) and by the Kruskall–Wallis method (intragroup comparisons). Correlations between postoperative pain and collateral swelling and collateral swelling and mouth opening were assessed with Pearson and Spearman methods.

## 3. Results

The patient demographics are presented in Table 1. No statistically significant differences between the groups were found regarding the mean age and the sex distribution.

There were statistically significant differences between the Er:YAG laser group and control group for postoperative pain on days 1, 3, 5, 7 and 10 (Table 2). Pain levels were significantly lower in the Er:YAG laser group than in the control group (*p* < 0.001) on all postoperative days (Table 2). Intragroup comparisons between days in both groups were also statistically different (*p* < 0.001) (Table 2).

On day 1, severe pain was experienced by 33.3% (10 patients) of the test group; moderately severe pain was also more frequently experienced by patients from the control group (*p* < 0.00001). Mild pain was experienced more often by the test group (29 patients (96.7%)) (*p* < 0.00001) (Table 3).

On day 3, absence of pain was observed in 10 (33.3%) patients in the test group, mild pain was experienced by 20 (66.7%) patients in the test group. Moderately pronounced pain was experienced by 20 (66.7%) patients from the control group, while strongly pronounced pain was experienced by 9 (30%) patients from the control group (Table 3).

On day 5, 25 (83.3%) patients in the test group felt no pain, mild pain was more pronounced in the control group (18 patients (60%)), than in the test group (5 patients (16.7%)), (*p* < 0.000557). In the control group, moderately severe pain was experienced by 11 (36.7%) patients, and severely severe pain was experienced by 1 (3.3%) patient (Table 3).

On day 7, 27 (90%) patients in the test group had no pain. Mildly pronounced pain was experienced by 28 (93.3%) patients in the control group than in the test group (3 patients (10%)) (*p* < 0.00001). Moderately severe pain was experienced by 2 (6.7%) patients in the control group (Table 3).

On day 10, 30 (100%) patients in the 1st group and 19 (63.3%) patients in the 2nd group had no pain. Mild pain was experienced by 11 (36.7%) patients in group 2 (Table 3).

There were statistically significant differences for postoperative swelling on days 1, 3, 5, 7 and 10 between the Er:YAG laser group and the control group (Table 4). The results in the Er:YAG laser group showed significantly lower collateral swelling levels than in the control group (*p* < 0.001) on all postoperative days, (Table 4). Intragroup comparisons between days in both groups were also statistically different (*p* < 0.001) (Table 4).

On day 1, mild collateral edema was more severe in the test group (28 patients (93%)) than in the control group (5 patients (16.67%)), respectively, *p* < 0.00001. Moderately severe swelling was less in the test group (2 patients (7%)) than in the control group (23 patients (76.67%)), respectively, *p* < 0.00001. Severe swelling in group 2 was expressed in 2 patients (6.67%) (Table 5).

On day 3 in the test group, the absence of collateral edema was observed in 12 patients (40%). Mild collateral edema was more pronounced in the test group (16 patients (53.33%)) than in the control group (1 patient (3.33%)), respectively, *p* < 0.000017. Moderately severe collateral edema was more pronounced in the control group (16 patients (53.33%)) than in the test group (2 patients (6.66%)), respectively, *p* < 0.00008. Severely marked collateral swelling in the control group was reported by 13 (43.33%) patients (Table 5).

On day 5, the absence of collateral edema in the test group was observed in 23 (90%) patients. Mild swelling was observed more in the control group (14 patients (46.67%)) than in the test group (5 patients (16.67%)), respectively, *p* < 0.012498. Moderately severe swelling was more pronounced in the control group (15 patients (50%)) than in the test group (2 patients (6.67%)), respectively, *p* < 0.000196. Severely pronounced collateral edema in the control group was observed in 1 patient (3.33%) (Table 5).

On day 7, an absence of collateral swelling was more observed in the test group (27 patients (90%)) than in the control group (4 patients (13.33%)), respectively, *p* < 0.00001. Mildly severe edema was more pronounced in the control group (24 patients (80%)) than in the test group (3 patients (10%)), respectively, *p* < 0.00001. Moderately severe edema was reported by 2 patients (6.67%) from group 2 (Table 5).

On day 10, the absence of collateral swelling was more observed in the test group (30 patients (100%)) than in the control group (19 patients (63.33%)). Weakly expressed collateral edema in the control group was observed in 11 patients (36.7%) (Table 5).

There were statistically significant differences for postoperative mouth opening on days 1, 3, 5, 7 and 10 between the Er:YAG laser group and the control group (Table 6). Results in the Er:YAG laser group showed significantly better results of mouth opening than in the control group (*p* < 0.001) on all postoperative days (Table 6). Intragroup comparisons between days in both groups were also statistically different (*p* < 0.001) (Table 6).

Correlation between postoperative pain and collateral swelling was analyzed in both groups on days 1, 3, 5, 7 and 10 (Table 7). In the laser group, the correlation between postoperative pain and collateral swelling was positive on all postoperative days; the correlation was statistically significant according to both the Pearson (R) and Spearman (RHO) correlation methods (*p* < 0.05). In the control group, the correlation between postoperative pain and collateral swelling was weakly positive on the 1st and 3rd days; while on the 5th, 7th, and 10th days, the correlation was negative according to both the Pearson (R) and Spearman (RHO) correlation methods (*p* > 0.05) (Table 7).

Correlation between postoperative collateral swelling and mouth opening was analyzed in both groups on days 1, 3, 5, 7 and 10 (Table 8). In the laser group, the correlation between postoperative collateral swelling and mouth opening was positive on all postoperative days, and this correlation was statistically significant according to both the Pearson (R) and Spearman (RHO) correlation methods (*p* < 0.05). In the control group, the correlation between postoperative collateral swelling and mouth opening was weakly positive on days 1 and 10; on days 3, 5 and 7, the correlation was negative according to both the Pearson (R) and Spearman (RHO) correlation methods (*p* > 0.05) (Table 8).

There were statistically significant differences of radiographic measures (RBH and RID) between groups after 12 weeks and 24 weeks after extraction (Table 9 and Table 10). The results of both radiographic measures (RBH and RID) in the Er:YAG laser group showed significantly better results of bone regeneration in the postoperative period than in the control group (*p* < 0.001) (Table 9). Intragroup comparisons between different periods in both groups were also statistically different (*p* < 0.001) (Table 9).

**Figure 6:**
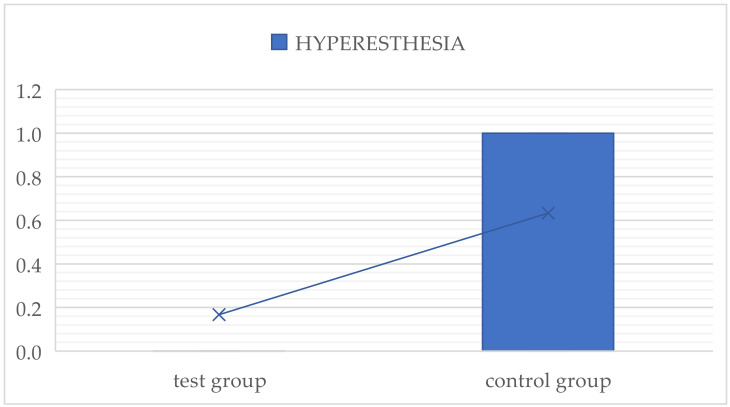
Intergroup comparison of hyperesthesia of the distal root of the second molar after third molar extraction.

Hyperesthesia of the distal root of the second molar after third molar extraction was more pronounced in the control group than in the test group (Figure 6).

## 4. Discussion

One of the achievements of modern surgical dentistry is the performance of surgical operations with high-intensity laser technologies, which allowed for improving the quality of performed operations, reducing traumatism and reaching a new level of laser surgery [35,36]. Erbium-based laser technologies have become widespread in oral surgery due to their ability to work on both soft and hard tissues, which is why the Er:YAG laser with the wavelength of 2940 nm can be applied in impacted third molars extraction [22,30,37].

Water is a chromophore: a substance capable of absorbing laser beam energy and transforming it into thermal energy. During laser action on tissues, water molecules boil and evaporate, thus increasing the intra-tissue pressure. Microbursts on the tissue surface occur, resulting in mechanical destruction (ablation effect) with minimal thermal changes [27,38,39,40]. Unlike other laser technologies, in addition to water molecules, the 2940 nm erbium laser is able to absorb calcium hydroxyapatite, which determines its effect on hard tissues and expands the possibilities of application in operations on bone and dental hard tissues [34,41,42,43].

A number of authors provide data on the acceleration of bone tissue regeneration at its alteration by erbium laser with a wavelength of 2940 nm [30,43]. According to densitometry data, bone density 3 months after cystectomy surgery performed with an erbium laser is greater than that of surgery performed with standard surgical instruments [31,33,44]. The radiation of an erbium laser with the wavelength of 2940 nm can accelerate the regeneration of periodontal tissues by increasing the proliferative activity of cellular structures. Erbium laser radiation stimulates the proliferation and migration of fibroblasts by inducing galectin-7 (LGALS7) protein. Galectin-7 is a protein involved in modulating intercellular interactions. These data help to understand the mechanism of action of laser radiation at the molecular level underlying the action of the Er:YAG laser on periodontal tissues [31].

The therapeutic and physiotherapeutic effect of an erbium laser with a wavelength of 2940 nm is able to suspend bone tissue resorption by applying laser radiation to the well after tooth extraction surgery, stimulating the processes of cell proliferation, enhancing the differentiation of stem cells, and accelerating the processes of tissue regeneration [39].

At present, more and more scientific papers are appearing on the topic of Er:YAG laser application in the surgery of removal of retained third molars, which have a positive effect of laser radiation including in the postoperative period [37,45,46]. It is known that the erbium laser with a wavelength of 2940 nm does not have an intense hemostatic effect like other laser technologies, which allows its use in the extraction of impacted teeth. Thus, the use of an Er:YAG laser allows to organize a good blood clot in the tooth cavity after extraction and provides visualization of the operative field [30,47].

A number of studies have presented comparative data on the use of the 2940 nm wavelength erbium laser and traditional methods of tissue alteration, such as scalpel and rotary instrument group in the operation of retained third molars extraction. In the postoperative period, a significant reduction in pain response, collateral edema and muscle contracture was observed during tooth extraction with the Er:YAG laser with a wavelength of 2940 nm in contrast to extraction with a rotary group of instruments and scalpel [32,37,43].

The use of an erbium laser with a wavelength of 2940 nm causes less traumatization of both soft tissues and bone tissue, which reduces the risk of postoperative complications. According to research data, patient satisfaction with the removal of third molars using an erbium laser was significantly higher than with the extraction using a scalpel and a rotary group of instruments. This is due to the fact that laser technology does not produce pronounced vibrations when working on bone tissue, thus ensuring a comfortable intraoperative period. Compared to traditional methods of tooth extraction, the use of laser technology in the extraction of impacted third molars provides a more comfortable intraoperative period and postoperative period [30].

Laser is a widely used therapy to avoid microbial infection and to stimulate wound healing through biological mechanisms [48,49,50]. There are some opinions about the ability of lasers to modulate the production of two β-defensins, hBD-1 and hBD-2 [51,52]. Defensins are believed to promote the activation of the immune response, following infections, through a chemotactic effect on monocytes [53]. β-defensins are represented by three distinct peptides, β-defensin 1, 2, and 3, and they are found in epithelial cells [54]. The functional roles of β-defensins are antimicrobial activity, immunomodulatory activity, regulating immune responses and the activation of immune cells, such as monocytes and dendritic cells [54]. Human β-defensin 2 (hBD-2) is primarily found in epithelial cells, particularly keratinocytes [53]. In addition to its antimicrobial activity, hBD-2 has been implicated in many physiological conditions associated with wound healing. Indeed, hBD-2 increases the production of proinflammatory cytokines and chemokines from keratinocytes and stimulates their proliferation and migration [51,55].

The limitations of this study are outlined below:

- Small number of patients;
- We did not take into account the different types of retention and tooth position;
- We did not divide patients by age and gender;
- A wide age range of patients (from 21 to 70 years);
- We included different numbers of men and women, and previous studies have shown different pain tolerance levels between men and women;
- We used the subjective method of swelling assessment in our study.

## 5. Conclusions

Postoperative pain and collateral swelling were less pronounced in the test group after extraction using an Er:YAG laser. Mouth opening in the test group was better than in the control group. Bone regeneration after impacted teeth extraction in the test group showed better results by assessing RBH and RID. The extraction of third molars using Er:YAG is less traumatic than with scalpel and rotation instruments.

## Figures and Tables

**Table 1:** Patient demographics.

Variables	Control Group	Test Group
Age, years (mean ± SD)	33.4 ± 5.62	37.1 ± 11.6
Median	33.5	34.0
Minimum	25	24
Maximum	40	56
Sex (n %)		
men	56.6%	60%
women	43.4%	40%

**Table 2:** Postoperative pain measurements (intergroup and intragroup comparisons).

Day	Er:YAG Group Mean ± SD Median IQR (Q3–Q1)	Control Group Mean ± SD Median IQR (Q3–Q1)	*p* Value (Mann–Whitney)
1	1.50 ± 0.78	5.03 ± 0.93	<0.001
	1.00	5.00	
	1.00 (2.00–1.00)	2.00 (6.00–4.00)	
3	0.93 ± 0.87	5.10 ± 0.96	<0.001
	1.00	5.00	
	1.00 (1.00–0.00)	2.00 (6.00–4.00)	
5	0.23 ± 0.57	3.33 ± 1.09	<0.001
	0.00	3.00	
	0.00 (0.00–0.00)	1.00 (4.00–3.00)	
7	0.10 ± 0.31	2.03 ± 0.89	<0.001
	0.00	2.00	
	0.00 (0.00–0.00)	1.75 (2.75–1.00)	
10	0.00 ± 0.00	0.37 ± 0.49	<0.001
	0.00	0.00	
	0.00 (0.00–0.00)	1.00 (1.00–0.00)	
*p* value (Kruskal–Wallis)	<0.001	<0.001	

**Table 3:** Severity of postoperative pain (intergroup comparisons).

Group	Day	0 Points	1–3 Points	4–5 Points	6–7 Points	8–9 Points	10 Points
Er:YAG group n (%)	1	-	29 (96.7%)	1 (3.3%)	-	-	-
	3	10 (33.3%)	20 (66.7%)	-	-	-	-
	5	25 (83.3%)	5 (16.7%)	-	-	-	-
	7	27 (90%)	3 (10%)	-	-	-	-
	10	30 (100%)	-	-	-	-	-
Control group n (%)	1	-	1 (3.3%)	19 (63.3%)	10 (33.3%)	-	-
	3	-	-	20 (66.7%)	9 (30%)	1 (3.3%)	-
	5	-	18 (60%)	11 (36.7%)	1 (3.3%)	-	-
	7	-	28 (93.3%)	2 (6.7%)	-	-	-
	10	19 (63.3%)	11 (36.7%)	-	-	-	-

**Table 4:** Postoperative collateral swelling measurements (intergroup and intragroup comparisons).

Day	Laser Group (Mean ± SD) Median IQR (Q3–Q1)	Control Group (Mean ± SD) Median IQR (Q3–Q1)	*p*-Value (Mann–Whitney)
1	1.40 ± 0.72	3.41 ± 0.83	<0.001
	1.00	4.00	
	1.00 (2.00–1.00)	1.0 (4.00–3.00)	
3	0.97 ± 1.10	4.41 ± 0.91	<0.001
	1.00	4	
	1.00 (1.00–0.00)	1.00 (5.00–4.00)	
5	0.367 ± 0.81	2.52 ± 0.99	<0.001
	0.00	3.00	
	0.00 (0.00–0.00)	1.00 (3.00–2.00)	
7	0.13 ± 0.43	1.21 ± 0.77	<0.001
	0.00	1.00	
	0.00 (0.00–0.00)	1.00 (2.00–1.00)	
10	0.00 ± 0.00	0.38 ± 0.49	<0.001
	0.00	0.00	
	0.00 (0.00–0.00)	1.00 (1.00–0.00)	
*p* value (Kruskal–Wallis)	<0.001	<0.001	

**Table 5:** Severity of postoperative collateral swelling (intergroup comparisons).

Group	Day	0 Points	1–2 Points	3–4 Points	5 Points
Laser group n (%)	1	_	28 (93%)	2 (7%)	_
	3	12 (40%)	16 (53.33%)	2 (6.66%)	_
	5	23 (76.67%)	5 (16.67%)	2 (6.67%)	
	7	27 (90%)	3 (10%)	_	_
	10	30 (100%)	_	_	_
Scalpel group n (%)	1	_	5 (16.67%)	23 (76.67%)	2 (6.67%)
	3	_	1 (3.33%)	16 (53.33%)	13 (43.33%)
	5	_	14 (46.67%)	15 (50%)	1 (3.33%)
	7	4 (13.33%)	24 (80%)	2 (6.67%)	_
	10	19 (63.33%)	11 (36.67%)	_	_

**Table 6:** Severity of postoperative mouth opening (intergroup and intragroup comparisons).

Mouth Opening						
Day	Er:YAG Group (Mean ± SD) Median IQR (Q3–Q1)	Control Group (Mean ± SD) Median IQR (Q3–Q1)	*p*-Value (Mann–Whitney) Between 1st and 2nd Groups	Δ Difference (Before and After Extraction) 1st Group	Δ Difference (Before and After Extraction) 2nd Group	*p*-Value (Mann–Whitney) Between Δ Difference 1st and 2nd Groups
Before Extraction	46.3 ± 4.69	45.0 ± 4.05	<0.435			
	47.0	45.0				
	6.25 (49.0–42.8)	5.75 (48.8–43.0)				
1	43.8 ± 4.34	37.7 ± 4.98	<0.001	2.57 ± 2.47	7.33 ± 4.09	<0.001
	44.5	37.0		2.00	6.00	
	5.25 (46.8–41.5)	7.00 (42.0–35.0)		1.00 (2.00–1.00)	2.75 (7.75–5.00)	
3	42.7 ± 5.07	33.4 ± 6.40	<0.001	3.60 ± 4.38	11.6 ± 5.88	<0.001
	44.0	34.0		2.00	10.0	
	8.25 (46.8–38.5)	9.00 (39.0–30.0)		2.00 (3.00–1.00)	3.75 (12.0–8.25)	
5	44.6 ± 4.61	35.1 ± 4.99	<0.001	1.73 ± 3.60	9.90 ± 4.48	<0.001
	46.0	35.0		0.00	10.5	
	7.50 (48.0–40.5)	8.50 (38.8–30.3)		1.00 (1.00–0.00)	2.00 (11.0–9.00)	
7	45.2 ± 4.46	41.0 ± 4.00	<0.001	1.10 ± 2.50	4.03 ± 3.51	<0.001
	46.5	41.5		0.00	2.00	
	6.25 (48.8–42.5)	6.75 (44.0–37.3)		0.750 (0.750–0.00)	2.75 (4.75–2.00)	
10	46.3 ± 4.63	42.3 ± 3.77	<0.001	0.0333 ± 0.183	2.73 ± 2.79	<0.001
	47.0	42.5		0.00	2.00	
	6.25 (49.0–42.8)	4.75 (44.8–40.0)		0.00 (0.00–0.00)	2.75 (3.75–1.00)	
*p* value (Kruskal-Wallis)	<0.009	<0.001		<0.001	<0.001	

**Table 7:** Intragroup correlation between postoperative pain and collateral swelling on days 1, 3, 5, 7 and 10.

	Laser Group Pearson (R); Spearman (RHO)	*p*-Value	Control Group Pearson (R); Spearman (RHO)	*p*-Value
day 1	R = 0.674	*p* < 0.001	R = 0.282	*p* = 0.065
	RHO = 0.472	*p* < 0.004	RHO = 0.238	*p* = 0.103
day 3	R = 0.576	*p* < 0.001	R = 0.032	*p* = 0.433
	RHO = 0.322	*p* < 0.041	RHO = −0.035	*p* = 0.574
day 5	R = 0.933	*p* < 0.001	R = −0.130	*p* = 0.752
	RHO = 0.839	*p* < 0.001	RHO = −0.149	*p* = 0.784
day 7	R = 0.937	*p* < 0.001	R = −0.061	*p* = 0.626
	RHO = 0.999	*p* < 0.001	RHO = −0.176	*p* = 0.824
day 10	_	_	R = −0.148	*p* = 0.783
	_	_	RHO = −0.148	*p* = 0.783

**Table 8:** Intragroup correlation between postoperative collateral swelling and mouth opening on days 1, 3, 5, 7 and 10.

	Laser Group Pearson (R); Spearman (RHO)	*p*-Value	Control Group Pearson (R); Spearman (RHO)	*p*-Value
day 1	R = 0.582	*p* < 0.001	R = 0.258	*p* = 0.084
	RHO = 0.580	*p* < 0.001	RHO = 0.217	*p* = 0.125
day 2	R = 0.751	*p* < 0.001	R = −0.214	*p* = 0.872
	RHO = 0.641	*p* < 0.001	RHO = −0.038	*p* = 0.580
day 5	R = 0.876	*p* < 0.001	R = −0.107	*p* = 0.713
	RHO = 0.776	*p* < 0.001	RHO = −0.163	*p* = 0.805
day 7	R = 0.528	*p* < 0.003	R = −0.093	*p* = 0.687
	RHO = 0.569	*p* < 0.001	RHO = −0.017	*p* = 0.535
day 10	-	-	R = 0.477	*p* = 0.004
	-	-	RHO = 0.527	*p* = 0.001

**Table 9:** Intragroup radiographic measures (radiographic infrabony defect (RID) in laser group and control group after extraction, 12 weeks and 24 weeks after extraction.

Radiographic Infrabony Defect (RID)			
Weeks	(Er:YAG) Laser Group (Mean ± SD) Median IQR (Q3–Q1)	Control Group (Mean ± SD) Median IQR (Q3–Q1)	*p* Value (Mann–Whitney)
0 weeks	8.10 ± 2.04	7.07 ± 2.30	0.067
	8.50	6.00	
	3.75 (10.0–6.25)	3.50 (8.75–5.25)	
12 weeks	3.03 ± 1.13	5.27 ± 2.00	<0.001
	3.00	5.00	
	2.00 (4.00–2.00)	2.00 (6.00–4.00)	
24 weeks	0.533 ± 0.629	3.17 ± 0.913	<0.001
	0.00	3.00	
	1.00 (1.00–0.00)	1.00 (4.00–3.00)	
*p* value (Kruskal–Wallis)	<0.001	<0.001	

**Table 10:** Intragroup radiographic measures (radiographic bone height (RBH)) in laser group and control group after extraction, 12 weeks and 24 weeks after extraction.

Radiographic Bone Height (RBH)			
Weeks	(Er:YAG) Laser Group (Mean ± SD) Median IQR (Q3–Q1)	Control Group (Mean ± SD) Median IQR (Q3–Q1)	*p* Value (Mann–Whitney)
0 weeks	7.03 ± 1.27	7.03 ± 1.27	1.000
	7.00	7.00	
	2.00 (8.00–6.00)	2.00 (8.00–6.00)	
12 weeks	12.2 ± 1.81	9.27 ± 1.34	<0.001
	12.5	9.00	
	2.75 (13.8–11.00)	1.75 (10.0–8.25)	
24 weeks	14.8 ± 1.32	11.1 ± 1.37	<0.001
	15.0	11.0	
	2.00 (16.0–14.0)	1.75 (12.0–10.3)	
*p* value (Kruskal–Wallis)	<0.001	<0.001	

## Data Availability

The raw data supporting the conclusions of this article will be made available by the authors on request.
